# Spatiotemporal Dynamics of Hemorrhagic Fever with Renal Syndrome, Beijing, People’s Republic of China

**DOI:** 10.3201/eid1512.081078

**Published:** 2009-12

**Keywords:** Hemorrhagic fever, renal syndrome, hantavirus, viruses, geographic information system, remote sensing, environmental factors, Beijing, China, dispatch

## Abstract

We used geographic information systems to characterize the dynamic change in spatial distribution of hemorrhagic fever with renal syndrome (HFRS) in Beijing, People’s Republic of China. The seasonal variation in its incidence was observed by creating an epidemic curve. HFRS was associated with developed land, orchards, and rice paddies.

Hemorrhagic fever with renal syndrome (HFRS), a rodent-borne disease caused by hantaviruses (family *Bunyaviridae*), is characterized by fever, acute renal dysfunction, and hemorrhage manifestations. Various rodent species are natural hosts and serve as sources of infection (*1*). Humans usually acquire hantavirus infection by contact with or inhalation of aerosols or excreta from infected rodents (*2*,*3*). In the People’s Republic of China, HFRS is caused mainly by 2 types of hantaviruses, Hantaan virus (HTNV) and Seoul virus (SEOV), each of which has co-evolved with a distinct rodent host. HTNV is associated with *Apodemus agrarius*, whereas SEOV, which causes a less severe form of HFRS, is associated with *Rattus norvegicus*. Although HFRS infection has long been recognized in many places throughout mainland China, HFRS was first reported in metropolitan Beijing in 1997. Since then, the natural foci have been established, and human cases were continuously diagnosed in the new disease-endemic region (*4*).

The presence and transmission of hantavirus depend on the distribution and infection of its animal hosts, which largely determine the incidence and extent of HFRS; such distribution and infection are usually determined by environmental elements (*5*,*6*). Ecologic studies in China demonstrated that elevation, precipitation, temperature, vegetation type, and soil type influenced transmission of HTNV (*7*,*8*). However, these studies were conducted on a relatively large scale, usually at the county or even province level. Environmental factors driving variability in HFRS incidence at a finer scale (e.g., township) remain poorly understood. The availability of detailed records of HFRS cases and environmental information in the newly established disease-endemic region provides an opportunity to explore possible factors underlying the emergence of the rodent-borne disease. In this study, we aimed to learn the current situation of endemic HFRS in Beijing, characterize its spatiotemporal distribution, and identify environmental factors possibly contributing to the incidence of the disease.

## The Study

The study area covered metropolitan Beijing (between 115°20′ and 117°30′E and 39°28′ and 41°05′N), including 220 townships of 18 districts with an area of 16,800 km^2^. The data on reported HFRS cases were obtained from the National Notifiable Disease Surveillance System, which included information about sex, age, residential address, and onset date of symptoms for each case.

**Figure 1:**
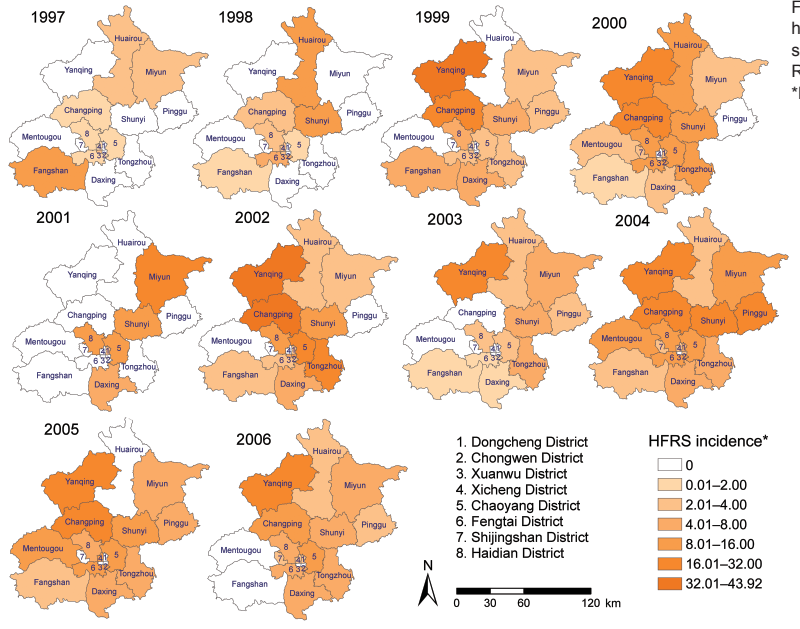
Yearly distribution of hemorrhagic fever with renal syndrome, Beijing, People’s Republic of China, 1997–2006. *Per 100,000 population.

A total of 852 HFRS cases were reported in Beijing metropolis during 1997–2006. The annual incidence of each district was calculated by using the fifth national census data in 2000 and mapped by using a geographic information system (GIS) technique by digitalizing village, street, and boundaries on the 1:100,000 topographic map of Beijing in ArcGIS 9.0 software (ESRI Inc., Redlands, CA, USA). Each HFRS case was geocoded according to residential address, and a layer including information about HFRS cases was created and overlayed on the digital map. By 2000, the disease had affected all the area of the city. However, the incidence in each district varied during the 10-year study period (Figure 1).

**Figure 2:**
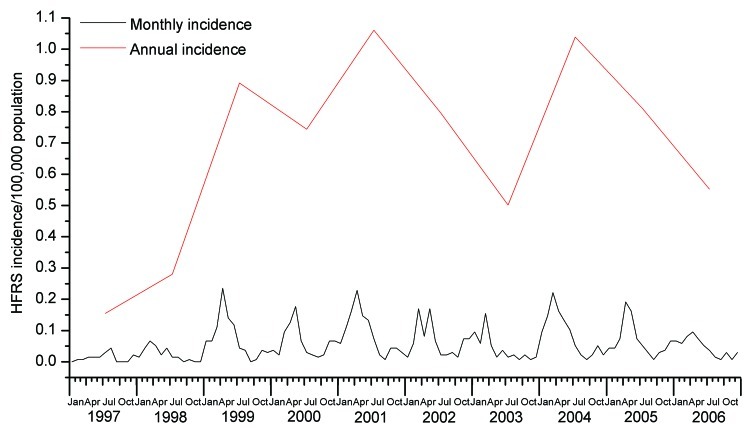
Temporal distribution of hemorrhagic fever with renal syndrome (HFRS), Beijing, People’s Republic of China, 1997–2006.

An epidemic curve was created to show the temporal distribution of HFRS in Beijing. The annual incidence had sharply increased from 1997 to 1999; thereafter it fluctuated around 0.8 ± 0.2/100,000 persons (Figure 2). Within each year, the incidence varied markedly; most cases occurred in winter and spring, usually peaking in April, although the disease was reported in almost every month (Figure 2).

To study factors related to HFRS spread, we compared the incidence with various environmental indicators. Data on elevation were collected from a digital elevation model. Land cover data were derived from 2005 Landsat 7 enhanced thematic mapper image by using ENVI 4.0 software (Research Systems Inc., Boulder, CO, USA). Land use types were classified as follows: built-up land, water body, dry land, scrub, orchard, irrigable land, rice paddies, and forest. Average elevation and area of different land use type of each township were calculated by ArcTools and Spatial Analyst module in ArcGIS 9.0 software (ESRI Inc.).

To determine the associations between the number of HFRS cases per township during the study period and elevation, as well as land use type, we performed standard Poisson regression analysis by using STATA 9.1 software (StataCorp LP, College Station TX, USA) (*9*). The percentage change in incidence in response to the value change of the variable by a given amount and its 95% confidence interval and corresponding p value were estimated after correction for overdispersion. The Poisson regression analysis indicated that built-up land, orchard, and rice paddies were significantly associated with HFRS at p<0.05 (Table). The incidence rose with increasing coverage of orchard and rice paddies, but dropped with decreasing coverage of built-up land.

**Table:** Poisson regression analysis of environmental factors in relation to HFRS incidence in Beijing, People’s Republic of China*

Variable (unit)†	% Change‡	95% CI	p value
Elevation (10 m)	−0.47	−15.9 to 17.8	0.96
Built-up land (1%)	−0.82	−1.50 to 0.14	0.02
Water body (1%)	−0.29	−5.88 to 5.63	0.92
Dry land (1%)	1.44	−0.21 to 3.12	0.09
Scrub (1%)	−1.26	−4.66 to 2.52	0.48
Orchard (1%)	4.33	1.71 to 7.00	<0.01
Irrigable land (1%)	1.22	−0.10 to 2.56	0.07
Rice paddies (1%)	27.8	4.4 to 56.3	0.02
Forest (1%)	0.60	−0.52 to 1.73	0.30

*HFRS, hemorrhagic fever with renal syndrome; CI, confidence interval. †Built-up land comprised human residences, industrial land, and land occupied by all kinds of roads. Water body comprised lakes, reservoirs, ponds, and watercourses of all kinds. Dry land comprised nonirrigated fields for planting crops. Scrub comprised bushes and shrubs. Orchards were areas producing fruits and raw materials for industry or for beverages. Irrigable land comprised fields under irrigation for planting crops. Rice paddies were fields for planting rice. Forest included areas with dense trees. ‡Percentage change in incidence rate if the value of the variable changed by the given amount.

## Conclusions

Since the first local infection was reported in 1997, HFRS cases have occurred in all 18 districts of Beijing, with a fairly stable annual incidence since 1999. The dynamic change in spatial distribution confirmed the focal nature of the rodent-borne disease. The seasonality is one of the epidemiologic characteristics of HFRS, and further indicates that HFRS in the region is caused by SEOV with domestic rats, with mainly *R. norvegicus* as the source of infection (*10*,*11*) The rat population apparently peaks in winter, resulting in a lagged impact on transmission and seasonal variation of HFRS. In addition, rats are more active in homes in the winter because of cold temperature outside; this increases the chance for humans to acquire the infection by contact or inhalation of aerosols and secretions from infected rodents.

Our findings suggest that residents of townships with not too much built-up land but with orchard or rice paddies are at highest risk for infection. The land-use variables (acting as economic development indicators) are likely to be suitable for predicting the presence and distribution of HFRS in Beijing, where the disease recently emerged and economic development has greatly increased. Suitable control measures, such as removing rodents and preventing them from entering houses and human food storage buildings, should be taken to reduce incidence in this new disease-endemic region. However, the emergence and endemicity of HFRS are not determined only by the economic development activities, although they are important environmental contributors to the transmission of the disease. Biologic, ecologic, and social factors such as population immunity level, abundance and infection rate of host rodents, and human behavior, also may affect transmission of HFRS. Further epidemiologic and ecologic studies are required to understand the exact variables contributing to the emergence and extension of the disease during urbanization.
